# Charged and Hydrophobic Surfaces on the A Chain of Shiga-Like Toxin 1 Recognize the C-Terminal Domain of Ribosomal Stalk Proteins

**DOI:** 10.1371/journal.pone.0031191

**Published:** 2012-02-15

**Authors:** Andrew J. McCluskey, Eleonora Bolewska-Pedyczak, Nick Jarvik, Gang Chen, Sachdev S. Sidhu, Jean Gariépy

**Affiliations:** 1 Department of Pharmaceutical Sciences, University of Toronto, Toronto, Ontario, Canada; 2 Sunnybrook Research Institute, Toronto, Ontario, Canada; 3 Banting and Best Department of Medical Research, Terrence Donnelly Center for Cellular and Biomolecular Research, University of Toronto, Toronto, Ontario, Canada; 4 Department of Medical Biophysics, University of Toronto, Toronto, Ontario, Canada; Institut Curie, France

## Abstract

Shiga-like toxins are ribosome-inactivating proteins (RIP) produced by pathogenic *E. coli* strains that are responsible for hemorrhagic colitis and hemolytic uremic syndrome. The catalytic A_1_ chain of Shiga-like toxin 1 (SLT-1), a representative RIP, first docks onto a conserved peptide SD[D/E]DMGFGLFD located at the C-terminus of all three eukaryotic ribosomal stalk proteins and halts protein synthesis through the depurination of an adenine base in the sarcin-ricin loop of 28S rRNA. Here, we report that the A_1_ chain of SLT-1 rapidly binds to and dissociates from the C-terminal peptide with a monomeric dissociation constant of 13 µM. An alanine scan performed on the conserved peptide revealed that the SLT-1 A_1_ chain interacts with the anionic tripeptide DDD and the hydrophobic tetrapeptide motif FGLF within its sequence. Based on these 2 peptide motifs, SLT-1 A_1_ variants were generated that displayed decreased affinities for the stalk protein C-terminus and also correlated with reduced ribosome-inactivating activities in relation to the wild-type A_1_ chain. The toxin-peptide interaction and subsequent toxicity were shown to be mediated by cationic and hydrophobic docking surfaces on the SLT-1 catalytic domain. These docking surfaces are located on the opposite face of the catalytic cleft and suggest that the docking of the A_1_ chain to SDDDMGFGLFD may reorient its catalytic domain to face its RNA substrate. More importantly, both the delineated A_1_ chain ribosomal docking surfaces and the ribosomal peptide itself represent a target and a scaffold, respectively, for the design of generic inhibitors to block the action of RIPs.

## Introduction

Shiga toxins such as Shiga-like toxin 1 (SLT-1) are produced by enteropathogenic *Escherichia coli* strains and represent the major cause of hemorrhagic colitis and hemolytic uremic syndrome [1], [2]. SLT-1 is a type II ribosome-inactivating protein (RIP) composed of a catalytically active A subunit non-covalently associated with a pentamer of B-subunits [3], [4]. This pentamer binds to the glycolipid globotriaosylceramide (CD77,Gb3), an event that leads to its internalization [5], [6], [7]. SLT-1 then traffics in a retrograde manner through the Golgi apparatus where it is proteolytically cleaved into an N-terminal catalytic A_1_ domain and a C-terminal A_2_ fragment non-covalently associated with its B-pentamer. Both A chain fragments remain linked by a single disulfide bond which is thought to be reduced in the ER lumen [8], [9], [10]. The A_1_ domain is then retrotranslocated to the cytosol by virtue of its newly exposed hydrophobic C-terminus, where it eventually docks onto ribosomes and subsequently depurinates a single adenine base (A^4324^) in the sarcin-ricin loop (SRL) of 28S rRNA [11], [12], [13], [14], [15]. This depurination event creates an apurinic site that prevents elongation factor 1 (EF-1)-dependent amino-acyl tRNA from binding to the ribosome and EF-2-catalysed translocation during elongation, leading to an inhibition of protein synthesis [16], [17], [18].

The protein component of the ribosome was first shown to contribute to the toxicity of RIPs when a 10^5^ fold increase in depurination rate was observed for ricin on native ribosomes when compared to protein-depleted ribosomes [19]. SLT-1 as well as other structurally and functionally related RIPs, require their docking to ribosomal proteins in addition to rRNA to maintain their optimal depurination rate and cytotoxic function [15], [19], [20], [21]. More recently, it has been revealed that the ribosomal protein components required for interacting with either type I (trichosanthin (TCS)) or type II (SLT-1 and ricin) RIPs are the ribosomal proteins RPP0, RPLP1 and RPLP2 (P0, P1, and P2) [15], [20], [22], [23]. These three proteins form the ribosomal stalk which is required for the binding of elongation factors leading to protein translation [24], [25], [26]. The eukaryotic stalk structure is composed of two heterodimers of the P1 and P2 proteins [27], [28], [29], which interact by virtue of the N-terminus of the P1 protein, at two specific locations on the P0 protein [30], [31], [32], [33], which subsequently binds to rRNA [34].

We have previously shown that the A_1_ chain of SLT-1 interacts with the ribosomal stalk proteins P0, P1, and P2 via a conserved C-terminal peptide (SDXDMGFGLFD, where X = D or E) [15]. In the present study, we demonstrate by yeast-2-hybrid (Y2H) and surface plasmon resonance (SPR) that the A_1_ chain of SLT-1 interacts with the C-terminal ribosomal stalk peptide with a micromolar dissociation constant. Specifically, the interaction of the A_1_ chain with the conserved C-terminal peptide SDDDMGFGLFD common to all three ribosomal stalk proteins exhibits a modest binding constant (K_d_ 13 µM), towards the monovalent peptide, with rapid *on* and *off* rates. This transient interaction is mediated by distinct charged and hydrophobic surfaces on the SLT-1 A_1_ chain, which are also essential for its full catalytic activity. Moreover, alanine-scanning mutagenesis revealed that anionic tripeptide and hydrophobic tetrapeptide motifs within the sequence SDDDMGFGLFD represent key anchor residues recognized by the A_1_ chain. These findings suggest that the nature of these interactions may play a guiding role in properly orientating RIP catalytic domains towards their substrate, the sarcin-ricin loop, and may represent a scaffold for the generation of RIP-specific antidotes.

## Methods

### Protein expression and purification

The wild-type SLT-1 was expressed as an N-terminal His_8_-tagged fusion construct in the *E. coli* strain JM101 (Agilent Technologies, Mississauga, ON), and purified as an AB_5_ holotoxin on nickel-NTA resin (Sigma-Aldrich, St. Louis, MO). The A_1_ chain was further purified from the holotoxin by first treating the purified AB_5_ variants with the protease furin (New England BioLabs, Ipswich, MA) and reducing the disulfide bond with 10 mM DTT. The A_1_ chain was then recovered on Nickel-NTA resin in the presence of a guanidine-HCl gradient to remove the untagged A_2_ chain and B subunits, followed by a re-folding step in PBS.

SLT-1 mutant variants corresponding to those that exhibited a lack of interaction as defined by Y2H screens (R172A, R176A, R179A, R188A, R176/179/188A, V191A, F226A, L233A, and S235A) were created by multi-step PCR using Taq polymerase. The first PCR step consisted of two reactions: (1) using sense primer 2 and one of the mutagenic antisense primers and (2) using an antisense primer 2 and one of the mutagenic sense primers (Table S1). The second step consisted of a single reaction using the previous PCR reactions as templates with sense and antisense primer 2 which allowed for the amplification of the entire mutated sequence including the incorporation of unique restriction endonuclease sites. These mutant gene sequences were digested with the appropriate restriction endonuclease, cloned into the *NheI* and *XhoI* sites of the pECHE10a vector (Molecular Templates Inc., Austin, Texas). The resulting SLT-1 A_1_ mutants were expressed and purified in the same manner as the wild-type SLT-1 A_1_ chain. The above-mentioned SLT-1 A_1_ variants used in subsequent experiments were judged by densitometry to be ≥85% pure (Figure S1).

### Peptide Synthesis

Synthetic peptides corresponding to the final 17, 11, and 7 residues of the C-terminal domain of ribosomal proteins P1 and P2, a control peptide, as well as all alanine-containing peptide variants of the final 11 residue peptide SDDDMGFGLFD used to measure binding affinities were assembled using the 9-fluorenylmethoxycarbonyl (Fmoc) method and Wang resin on a PS3 Peptide Synthesizer (Protein Technologies Inc., Tucson, AZ). Fmoc-Asp(OMpe)-OH (NovaBiochem, Gibbstown, NJ) and Fmoc-Glu(OBt)-Ser(ΨMe, MePro)-OH (NovaBiochem) dipeptide were used to avoid aspartimide formation. The Fmoc protecting groups were removed using 20% Piperidine/0.1 M HOBt in DMF during synthesis. The N-α-amino group (N-terminus) of the peptides was labeled overnight with biotin using a ten-fold excess of biotin (Molecular Probes, Burlington, ON) in the presence of HCTU/HOBt and DIPEA. Biotinylated peptides were cleaved from their support using 3 mL of a TFA/TIS/EDT/Water (92.5∶2.5∶2.5∶2.5%) mixture for 4 hrs at RT and purified by HPLC on a C_18_ semi-preparative column using an acetonitrile gradient from 5% to 100% in 20 min. Peptide masses were then confirmed by mass spectrometry.

### Yeast-2-Hybrid

Yeast ribosomes are rapidly inactivated by the A chains of RIPs such as SLT-1. A catalytically inactive form of the A_1_ chain (CIA_1_) was thus generated by introducing two mutations, namely E167A and R170A, within its catalytic region [11], [15]. These two point mutations decrease the toxicity of the A_1_ chain by 10,000 fold, therefore enabling yeast to grow during the expression of the A_1_ chain of SLT-1 [35], [36]. The yeast-2-hybrid (Y2H) technique [37], takes advantage of the GAL4 transcription factor that can be spliced into 2 complementary domains: a DNA binding domain (DNA-BD) and a transcription activation domain (AD). When pairs of bait/prey proteins interact, the GAL4 DNA-BD and transcription AD modules are assembled leading to the activation of survival genes (i.e.: HIS3).

The CIA_1_ SLT-1 gene sequence was used as a template to construct several charged and hydrophobic single point mutations to alanine by multistep PCR using sets of mutagenic primers (Table S1). The CIA_1_ as well as the point mutants were cloned into the bait vector pGBKT7 (Clontech, Mountain View, CA) between the *NdeI* and *BamHI* sites and expressed as fusions to the C-terminus of the GAL4 DNA-binding domain (GAL4 DNA-BD). The human gene sequence corresponding to RPLP2 (P2) was cloned into the prey vector pGADT7 (Clontech) and expressed as a fusion construct to the GAL4 activation domain (GAL4-AD). The pGBKT7-CIA_1_ or one of the charged or hydrophobic point mutants were co-transformed with pGADT7-P2 separately into the yeast strain AH109 [*MATa*, *trp1-901*, *leu2-3*, *112*, *ura3-52*, *his3-200*, *gal4*Δ, g*al80*Δ, *LYS2::GAL1_UAS_-GAL1_TATA_-HIS3*, *GAL2_UAS_-GAL2_TATA_-ADE2*, *URA3::MEL1_UAS_-MEL1_TATA_-lacZ*, *MEL1]* (Clontech).

Transformed cells were plated onto SD agar lacking Trp and Leu (−Trp/−Leu), to select for the presence of both plasmids, and incubated for 72 h at 30°C. A single colony of each transformation was then inoculated into SD −Trp/−Leu broth and shaken at 30°C overnight. Overnight cultures were centrifuged at 4000 rpm for five minutes followed by washing and equilibration to OD_600_ of 1.0 in phosphate-buffered saline (PBS). Samples were then serially diluted 10-fold followed by spotting onto SD agar −Trp/−Leu to select for the presence of both plasmids and SD −Trp/−Leu/−His to select for an interaction between the two proteins. Plates were incubated for 72 h at 30°C.

### Surface Plasmon Resonance Measurements

Toxin-peptide binding affinities were assessed by surface plasmon resonance (SPR) using a ProteOnXPR36 array biosensor [38] (Bio-Rad) in PBS buffer at 25°C. A neutravidin (NLC) sensor chip (Bio-Rad) was preconditioned by four pulses of 1 M NaCl, 50 mM NaOH, and 100 mM HCl, which was followed by an equilibration step in PBS. Biotinylated peptides corresponding to the final 17, 11, and 7 residues of the conserved peptide and a control peptide with no sequence homology were immobilized on the NLC sensor chip followed by a wash step in 1 M NaCl, to remove unbound peptide and a PBS wash to equilibrate the chip. Purified SLT-1 wild-type A_1_ chain was tested at 10 different concentrations in quadruplicate beginning with 30 µM and diluting 2-fold in PBS to 60 nM, followed by an injection of PBS alone. The wild-type A_1_ chain was exposed to all ribosomal peptide variants. Each concentration of SLT-1 A_1_ chain variant was exposed to the NLC chip harboring the ribosomal peptides at a flow rate of 50 µl/min for 60 s with a dissociation time of 120 s. A 1 M NaCl wash step was performed for 18 s at a flow rate of 100 µl/min following each protein concentration. The chip was then equilibrated with PBS at a flow rate of 100 µl/min for 120 s.

**Figure 1:**
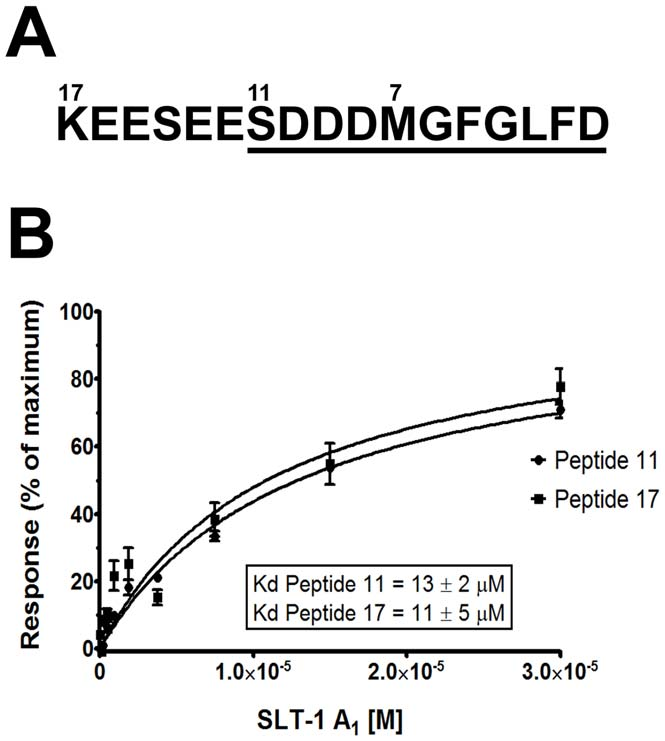
The A_1_ chain of SLT-1 binds to a conserved C-terminal ribosomal peptide. (A) Amino acid sequence representing the 17-residue C-terminus common to ribosomal stalk proteins P1 and P2. The last 11 amino acids (underlined) delimit the shortest peptide element shown to interact with the A_1_ chain [15]. (B) Relative surface plasmon resonance (SPR) signals for the A_1_ chain of SLT-1 binding to immobilized, biotinylated monomeric synthetic peptides were plotted as a function of SLT-1 A_1_ chain concentration. The calculated dissociation constants (K_d_) suggest that both monomeric peptides have similar affinities for the A_1_ chain. Each point on the curve represents the average relative SPR signals from experiments performed in quadruplicate.

Additional SPR experiments measuring (i) the affinities of charge and hydrophobic A_1_ chain mutants, (ii) the contributions of electrostatic interactions by increasing salt concentrations, and (iii) to establish the anchor residues within the conserved ribosomal stalk peptide, were performed in triplicate using the same protocol described above. Only the final 11 residues of the conserved peptide KEESEESDDDMGFGLFD were used, as the removal of the six N-terminal residues had no effect on binding and therefore do not contribute significantly to the interaction (Figure 1).

**Figure 2:**
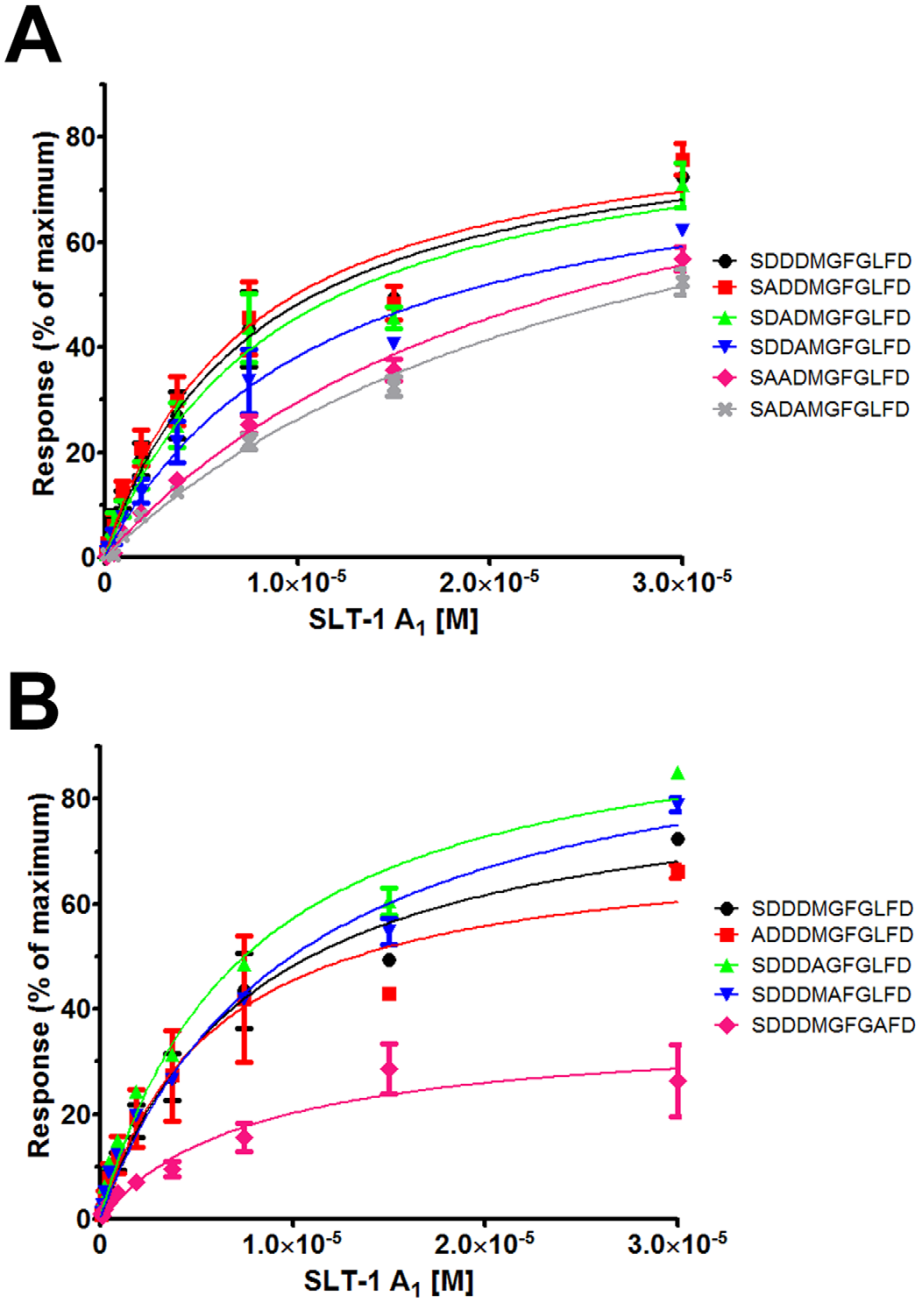
Surface plasmon resonance analysis of alanine-containing peptide variants of the conserved C-terminal ribosomal stalk peptide SDDDMGFGLFD confirms that the interaction with the A_1_ chain of SLT-1 requires both electrostatic and hydrophobic contacts. The peptide sequence corresponding to the final 11 residues of the conserved C-terminal peptide (SDDDMGFGLFD) was substituted at each position for an alanine residue. Individual peptides corresponding to a substitution of charged (Panel A) or other residues (Panel B) were biotinylated and immobilized on an NLC SPR sensor chip. Each monomeric peptide was exposed to ten 2-fold serial dilutions of the A_1_ chain of SLT-1 in triplicate and the responses were subtracted from buffer alone and a control peptide. The SPR responses for the single and double/triple alanine variants were graphed and compared to the control natural peptide. Amino acid substitutions that resulted in a peptide that lacked an interaction with the A_1_ chain of SLT-1 could not be plotted. Calculated dissociation constants are reported in Table 1.

**Figure 3:**
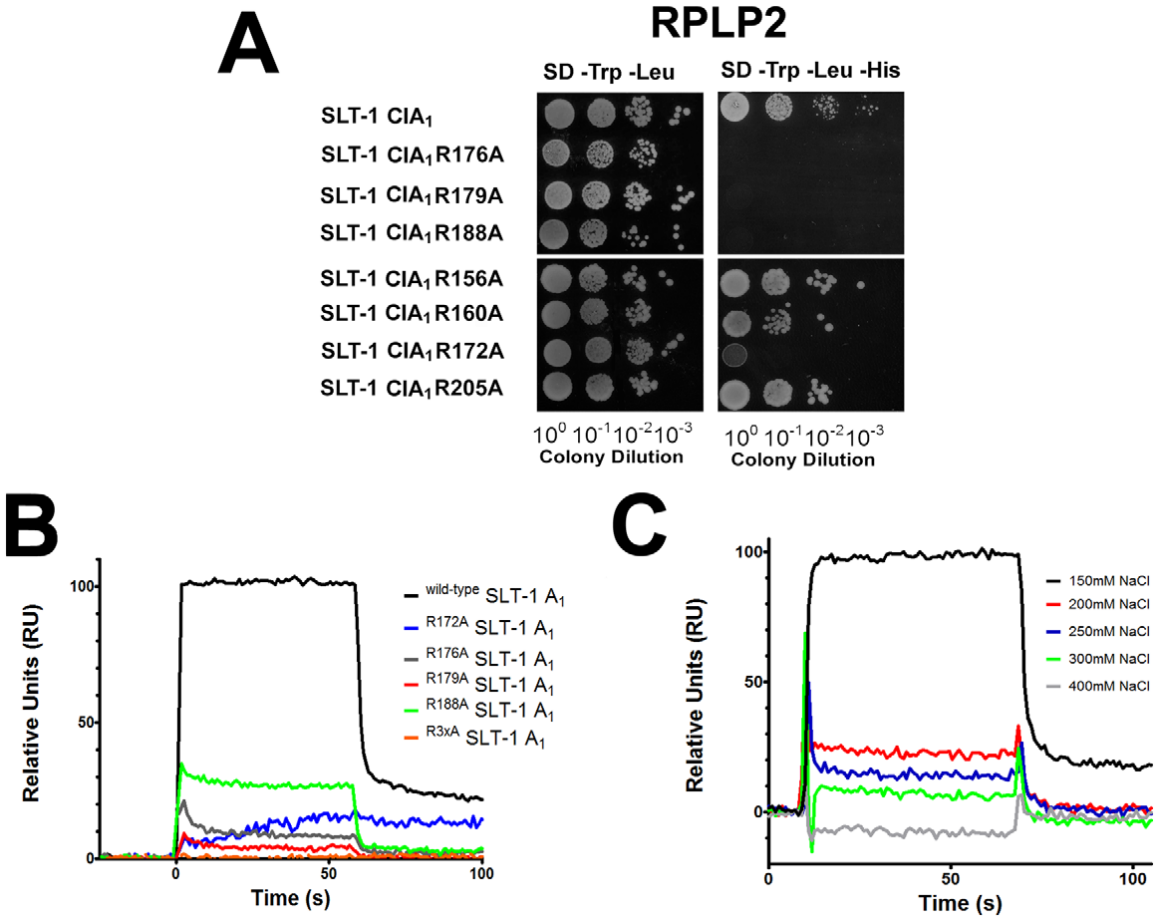
The A_1_ chain of SLT-1 harbors a cationic surface composed of a cluster of arginine residues that interact with the ribosomal stalk protein P2 and the conserved C-terminal peptide. (A) A vector expressing a catalytically inactive variant of the SLT-1 A_1_ domain (CIA_1_) or one of the arginine-to-alanine point mutants as fusion partners with the GAL4 DNA-BD domain were co-transformed in the yeast strain AH109 with a vector expressing ribosomal protein P2 as a fusion construct to the GAL4-AD. The transformed yeast cells were plated on SD agar −Trp/−Leu. The resulting yeast colonies were grown overnight, and spotted (10 µl) as 10-fold serial dilutions onto SD medium lacking Trp and Leu to select for the presence of each plasmid followed by spotting on SD media lacking Trp, Leu, and His to select for interacting partners leading to colony growth. (B) SPR profiles illustrating the decrease in relative units for the arginine-to-alanine SLT-1 A_1_ chain variants in relation to the wild-type A_1_ chain, at a concentration of 15 µM, when presented to the immobilized peptide SDDDMGFGLFD. (C) Increasing salt concentrations led to a decrease or loss of binding of wild-type SLT-1 A_1_ chain when exposed to the peptide SDDDMGFGLFD. SPR traces were plotted for the wild-type SLT-1 A_1_ chain (15 µM) as a function of increasing salt concentrations.

Equilibrium data was used to calculate peptide binding constants to the A_1_ chain in light of fast *on-* and *off*-rates. In all cases, the response data given from the ribosomal peptides was subtracted from the control peptide as well as PBS alone to eliminate non-specific binding. The resulting data for each concentration was averaged and plotted as percent response units (% RU) versus A_1_ chain concentration using GraphPad Prism® (Figure 1). In the cases where binding affinities could not be calculated, due to a decrease or loss of binding, raw data was plotted as relative units (RU) compared to the same concentration (15 µM) of wild-type toxin A_1_ chain in the same buffer (Figures 2B, 2C, 3B, and 3C).

### Protein Synthesis Assay

T7-coupled transcription-translation (TnT) reticulocyte lysate assays (Promega, Madison, WI) were performed in order to determine if a decrease in ribosomal stalk binding altered the toxicity profiles of SLT-1 A_1_ chain point mutants when compared to the wild-type toxin. TnT assays were performed, according to the manufacturer's instructions, using eight 10-fold serial dilutions of the wild-type or SLT-1 A_1_ variants in PBS (starting with 1 µM). Protein synthesis was measured using a luciferase reporter plasmid (500 µg) through the incorporation of [^35^S]-methionine (10 µCi; GE Healthcare, Piscataway, NJ) after a 90 min incubation at 30°C. Samples (20 µl) were loaded on a gradient (4–12%) SDS PAGE gel, and labeled protein bands revealed using a Storm® Phosphorimager (GE Healthcare). The addition of PBS alone was used as a control.

## Results

### The catalytic A_1_ chain of SLT-1 binds to the conserved C-terminal ribosomal peptide

We have previously shown that the A_1_ chain of SLT-1 binds to the conserved peptide KEESEESD(D/E)DMGFGLFD found at the C-terminus of ribosomal stalk proteins P0, P1 and P2 [15]. However, the molecular details of this interaction are unknown and could provide new evidence surrounding the mechanism of ribosome-inactivation by RIPs. The binding of the conserved C-terminal domain of the ribosomal proteins P1 and P2 to the wild-type SLT-1 A_1_ chain was thus analyzed by surface plasmon resonance (SPR). Biotinylated peptides corresponding to the final 17, 11 and 7 residues of ribosomal stalk proteins P1 and P2 were immobilized on an sensor chip (NLC; Bio-Rad, Hercules, CA) and each peptide ligand was exposed to increasing concentrations of the wild-type SLT-1 A_1_ chain. In light of the fast association and dissociation times observed for this binding event, equilibrium data from four independent experiments were instead used to calculate the dissociation constants of peptides binding to the A_1_ chain. It was determined that the A_1_ chain of SLT-1 interacted with the C-terminal 17 (KEESEESDDDMGFGLFD) and 11 residues (SDDDMGFGLFD) of P1 and P2 with comparable binding affinities of 11±5 µM and 13±2 µM respectively (Figure 1). The final 7 residues (MGFGLFD) did not bind to wild-type SLT-1 A_1_ chain (data not shown), as previously reported by pull-down experiments [15].

### Delineating key residues within the ribosomal peptide recognized by the SLT-1 A_1_ chain

The SLT-1 A_1_ chain docks within the ribosomal stalk by binding to specific residues within the peptide sequence SDDDMGFGLFD. A series of synthetic peptides containing alanine substitutions were generated by solid-phase peptide synthesis, to establish which residues within the peptide motif were important for its interaction with the A_1_ chain of SLT-1. Specifically, alanine was introduced at each position within the peptide SDDDMGFGLFD. These peptides were modified at their N-terminus with biotin, immobilized on an NLC sensor chip (BioRad), and exposed to graded concentrations of the SLT-1 A_1_ chain. The SPR equilibrium data was collected, in triplicate, and responses were plotted as a function of A_1_ chain concentration to calculate dissociation constants. It was determined that mutations of any of the five C-terminal residues to alanine (SDDDMGFGLFD) resulted in a decrease or complete loss of binding to the A_1_ chain. (Table 1 and Figure 2). Interestingly, individual mutations of the N-terminal, negatively charged aspartyl residues to alanine did not show any significant effect on the affinity suggesting that such interaction with the A_1_ chain may require the removal of more than one aspartic acid residues within the DDD tripeptide regardless of their position (Figure 2A). Therefore, the contribution of the charged aspartic acid residues within the peptide was further assessed through the generation of double and triple aspartate-to-alanine SLT-1 A_1_ chain mutants. It was observed that mutations to any two aspartic acid residues to alanines, particularly involving residues at positions 3 and 4 caused at least a two-fold decrease in binding (Table 1 and Figure 2A). This comprehensive binding analysis confirmed that both the charge and hydrophobic elements (Figures 2A and B, respectively) of the conserved peptide SDDDMGFGLFD are required for the optimal binding of the A_1_ chain of SLT-1 to the ribosomal stalk.

**Table 1:** Binding of synthetic, alanine-containing peptide variants of the ribosomal stalk peptide SDDDMGFGLFD to the A_1_ chain of SLT-1 as measured by surface plasmon resonance.

Peptide	K_d_ (µM)
SDDDMGFGLFD	10.6±2.4
ADDDMGFGLFD	12.7±4.3
SADDMGFGLFD	9.75±2.4
SDADMGFGLFD	11.6±2.8
SDDAMGFGLFD	15.9±3.5
SDDDAGFGLFD	7.5±0.2
SDDDMAFGLFD	9.9±0.5
SDDDMGAGLFD	≥50
SDDDMGFALFD	≥50
SDDDMGFGAFD	41±11
SDDDMGFGLAD	≥50
SDDDMGFGLFA	≥50
SAADMGFGLFD	23.9±1.6
SADAMGFGLFD	28.2±1.9
SDAAMGFGLFD	≥50
SAAAMGFGLFD	≥50

Dissociation constants (K_d_) were calculated from curves relating changes in relative surface plasmon resonance signal observed as a function of SLT-1 A_1_ concentration (Figure 2). Each K_d_ value was derived from an average of three experiments. A K_d_ value of ≥50 µM was assigned to peptides displaying no measurable binding to the A_1_ chain of SLT-1.

### The binding between the SLT-1 A_1_ chain and the conserved peptide SDDDMGFGLFD involves electrostatic interactions

In view of the negatively charged nature of the conserved ribosomal C-terminal peptide (aspartic acid [D] residues), we investigated whether a complementary positively charged surface was present on the A_1_ chain of SLT-1 that would promote electrostatic interactions with this ribosomal peptide. Surface-exposed arginine residues were mutated to alanine (R-A) in order to assess their importance as anchoring residues for the conserved ribosomal stalk peptide. SLT-1 A_1_ domain constructs with point mutations at arginine residues were generated by PCR using a catalytically-inactive SLT-1 A_1_ chain (CIA_1_) gene for their non-lethal expression in yeast. These R-A mutants were assessed for their ability to bind the full-length P2 protein in a yeast two-hybrid (Y2H) assay where co-transformation of each mutant with P2 were then serially diluted on medium which selected for an interaction between the two proteins (SD −Trp/−Leu/−His). Alanine mutations at residues R172, R176, R179, and R188 in the A_1_ domain led to a loss of growth, implying that these arginines interact with the ribosomal peptide (Figure 3A). Alanine mutations at other arginine sites within the A_1_ domain did not affect yeast survival suggesting that they are not involved in ribosomal docking (Figure 3A). Importantly, residues R172, R176, R179, and R188 are clustered on the surface of the A_1_ chain suggesting the existence of a complementary positively charged surface within the A_1_ chain.

To further define the effect of charged residues on the binding strength of A_1_ chain to the peptide SDDDMGFGLFD, we expressed and purified recombinant SLT-1 A_1_ chain variants, which showed a loss of interaction with the P2 protein by Y2H (R172A, R176A, R179A, R188A, and R176/179/188A), to directly assess their binding affinities towards this peptide by SPR. As expected, the binding affinity of the SLT-1 A_1_ chain R-A mutants for the peptide (≥50 µM, no measurable binding) was lower than the affinity observed for the wild-type toxin (K_d_ = 13 µM). The SLT-1 R188A A_1_ chain mutant was the only variant with a measurable binding constant (approximately 50 µM), while the other SLT-1 A_1_ chain variants (R172A, R176A, R179A, and R176/179/188A) lacked detectable SPR responses even at high concentrations and were determined to be weaker than 50 µM (Figure 3B), In addition, when the concentration of salt in the running buffer (PBS) was increased, the interaction between the wild-type SLT-1 A_1_ chain and the peptide SDDDMGFGLFD was decreased, further confirming the importance of electrostatic interactions (Figure 3C).

### Hydrophobic interactions also contribute to binding the conserved C-terminal domain of ribosomal stalk proteins

**Figure 4:**
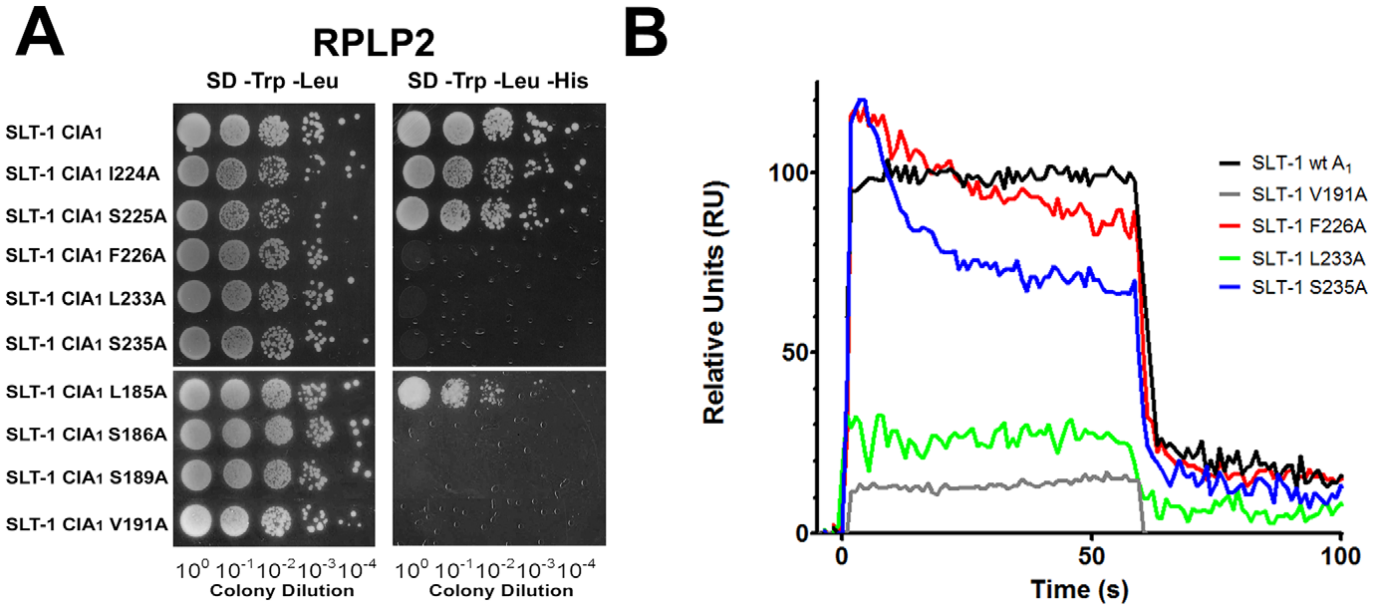
The interaction of the A_1_ chain of SLT-1 with the ribosomal stalk protein P2 and the C-terminal peptide SDDDMGFGLFD also involves hydrophobic residues within the A_1_ chain. (A) Bait vectors expressing either a catalytically inactive variant of the wild-type SLT-1 A_1_ domain (CIA_1_) or one of the hydrophobic mutants were co-transformed in the yeast strain AH109 with a prey vector expressing ribosomal protein P2. The transformed yeast cells were plated on SD agar −Trp/−Leu. The resulting yeast colonies were grown overnight, and spotted (10 µl) as 10-fold serial dilutions onto SD medium lacking Trp and Leu to select for the presence of each plasmid followed by spotting on SD media lacking Trp, Leu, and His to select for interacting partners. (B) SPR profiles (plotted at 15 µM) demonstrate that hydrophobic mutants F226A and S235A in the SLT-1 A_1_ chain have a minor effect on the binding to the conserved peptide SDDDMGFGLFD and the SLT-1 V191A and L233A A_1_ chain mutants cause a drastic decrease in binding. Experiments were performed in triplicate.

The presence of a hydrophobic tetrapeptide motif (SDDDMGFGLFD) led us to hypothesize that a hydrophobic patch on the A_1_ chain of SLT-1 may also contribute to its interaction with the ribosomal stalk peptide. To test this hypothesis, several hydrophobic and serine residues namely, L185, V191, I224, S225, F226, L233, and S235 on the surface of the A_1_ chain that are in the vicinity of the previously defined arginine cluster were mutated to alanine residues. These point mutants were generated by PCR using a catalytically-inactive SLT-1 A_1_ chain (CIA_1_) gene serving as a template in order to subsequently express them as non-toxic variants in yeast. Their binding to the ribosomal protein P2 was examined by Y2H (Figure 4A). Yeast transformations were serially diluted on SD-media lacking Trp, Leu, and His, which selects for the interaction between the two proteins. It was determined that residues V191, F226, L233, and S235 are important for the A_1_ chain – ribosomal peptide interaction, while residues L185, I224 and S225 do not participate in these interactions (Figure 4A). The contribution of the hydrophobic and serine residues located on the surface of the A_1_ chain was investigated by SPR to confirm the interactions of recombinant A_1_ chain variants with the C-terminal peptide SDDDMGFGLFD. It was found that the V191A and L233A variants exhibited a large decrease in binding while variants harboring either a F226A or a S235A mutation displayed different binding kinetics and binding affinities of 3 and 5 µM, respectively. These affinities are comparable to that of wild type A_1_ chain and may be attributed to the altered binding kinetics (Figure 4B and Figure S2).

### Altering the cationic or hydrophobic surfaces of the catalytic domain of SLT-1 is sufficient to lower its ability to inhibit protein synthesis

We have shown by both Y2H and SPR that positively charged and hydrophobic residues located on the surface of the A_1_ chain form cationic and hydrophobic patches allowing for its binding to ribosomal stalk proteins via the C-terminal peptide SDDDMGFGLFD. These interactions were measured as monomeric events, meaning that such interactions and affinity constants reflect complexes involving a single ribosomal peptide binding to a single SLT-1 A_1_ chain. In reality, the peptide SDDDMGFGLFD is repeated five times within the context of the ribosomal stalk suggesting that even a dissociation constant in the low µM range could be significant due to the higher concentration of low affinity targets that could maintain the docking of the A_1_ chain to the stalk.

**Figure 5:**
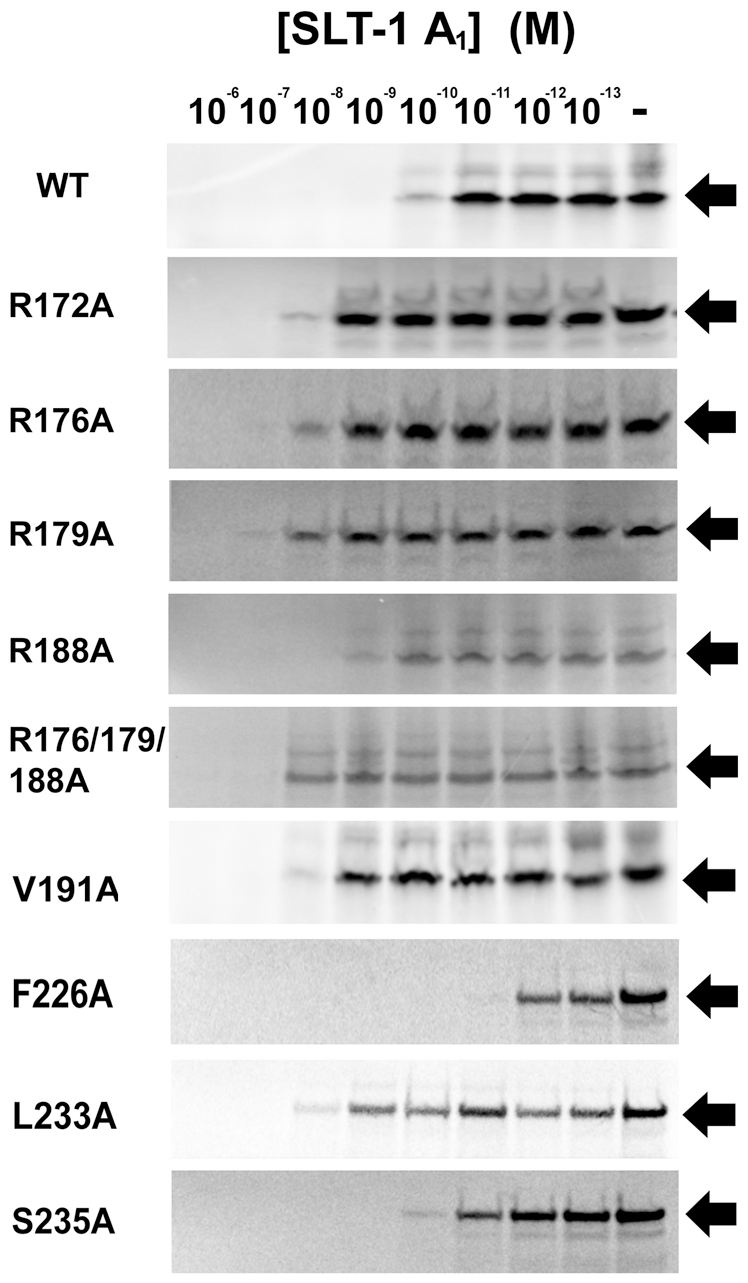
Arginine-to-alanine and hydrophobic variants of SLT-1 A_1_ that bind weakly to the monomeric conserved C-terminal motif display altered ribosome-inactivating activities when compared to the wild-type A_1_ chain. Eight ten-fold serial dilutions of the wild-type and each charge and hydrophobic A_1_ chain variant was dispensed into an *in vitro* transcription and translation-coupled rabbit reticulocyte lysate system to monitor their ability to block protein synthesis (methods section). The level of *in vitro* protein synthesis was assessed by measuring the incorporation of [^35^S]-methionine into the reporter protein luciferase during its synthesis. The expression of radiolabeled luciferase (arrow) was then resolved by SDS-PAGE and quantified using a phosphorimager. The addition of PBS alone (- lane) was used as a control.

To determine the effects of avidity on the binding and subsequent cytotoxicity of the A_1_ chain of SLT-1, we further tested whether the charged or hydrophobic residues that are responsible for the interaction with the stalk peptide were also required for its full effect on protein synthesis inhibition. It was thus hypothesized that decreasing the affinity of the A_1_ chain to the ribosomal stalk proteins by introducing arginine-to-alanine (R-A) or hydrophobic-to-alanine mutations, which have previously been shown to perturb the interaction, would result in a decrease in its ability to inhibit protein synthesis when compared to the wild-type A_1_ chain. To test this hypothesis, ten-fold serial dilutions of each A_1_ chain variant (R172A, R176A, R179A, R188A, R176/179/188A, V191A, F226A, L233A, and S235A) were added to a T7-coupled rabbit reticulocyte lysate transcription-translation system to quantify their ability to block the biosynthesis of [^35^S]-methionine-labeled luciferase. As predicted, A_1_ chain mutants that exhibited a drastic decrease in affinity for the ribosomal stalk peptide (namely R172A, R176A, R179A, R188A, R176/179/188A, V191A, and L233A) displayed an increase in protein expression as compared to the wild-type toxin (Figure 5). These findings suggest that the binding of the A_1_ chain to the ribosomal stalk, in the context of a functional eukaryotic ribosome, correlates with its ability to inhibit protein synthesis with all SLT-1 A_1_ chain mutants able to block protein synthesis at high concentrations. As expected, the S235A mutant did not significantly alter cytotoxicity profiles when compared to the wild type A_1_ chain. The translation inhibition effects were also smaller for the R188A mutant, which can be attributed to the retention of approximately 30% of the binding compared to the wild-type toxin (Figures 3B and 5). Interestingly, the F226A mutation displayed an increase in catalytic activity as measured by TnT, an observation that is currently under investigation (Figure 5).

## Discussion

The catalytic domains of ribosome-inactivating proteins (RIPs) such as SLT-1, ricin, and TCS [15], [20], [22], [23], [39] have been shown to interact with the eukaryotic ribosomal stalk, a heteropentamer composed of two heterodimers of the proteins RPLP1 (P1) and RPLP2 (P2) non-covalently associated to the protein RPP0 (P0) [27], [28], [29], [30], [31], [32], [33]. One known docking site for these catalytic chains on ribosomes is through their binding to a C-terminal peptide KEESEESDXDMGFGLFD (where X is either D or E) encoded by all three stalk proteins P0, P1, and P2. The pentavalent display of this peptide sequence within the stalk provides in theory up to five docking sites for RIP catalytic chains such as the A_1_ chain of SLT-1 to orient themselves on ribosomes, an event that may facilitate ribosome depurination leading to the inhibition of protein synthesis and apoptosis. The presence of five copies of this peptide motif suggests that valency is a critical factor and that the peptide-RIP interaction may be transient and of low affinity. In the present study, we have established by surface plasmon resonance that the A_1_ chain of SLT-1 binds to the conserved monomeric 17-residue long C-terminal peptide (KEESEESDDDMGFGLFD) and its truncated 11-residue form (SDDDMGFGLFD) with comparable modest affinities (K_d_ of 11±5 µM and 13±2 µM, respectively) (Figure 1). Interestingly, the *on-* and *off*-rates could not be measured for the A_1_ chain interacting with the monomeric peptide and such fast association and dissociation rates were expected since the binding of other RIPs namely saporin, restrictocin, and ricin to ribosome also show unusually fast kinetics and catalytic efficiencies [39], [40], [41], [42]. The rapid *on-* and *off*-rates are plausible in light of the fact that the interaction between RIPs and the ribosome must be a transient event in order for one ricin molecule to depurinate ∼2000 mammalian ribosomes/min and for a single SLT-1 A_1_ toxin molecule being required to enter the cytosol to elicit cell death [43], [44].

A previous study by our group had indicated that the C-terminal peptide inhibits the catalytic activity of the SLT-1 A_1_ chain in blocking protein synthesis in an *in vitro* transcription-translation assay (McCluskey *et al.*, 2008). This finding suggested that such a peptide may serve as a template in designing a new class of inhibitors able to block the action of RIPs. We thus further analyzed the key components of this peptide involved in its interaction with SLT-1 A_1_ chain. Specifically, synthetic peptide analogues of this sequence were used to define that the anionic tri-aspartyl sequence DDD and the four C-terminal residues FGLF within the C-terminal peptide (SDDDMGFGLFD) represent the two major anchors that interact with complementary cationic (Figure 2A) and hydrophobic (Figure 2B) surfaces on SLT-1 A_1_ chain. As in the case of most ER-routed toxins, the A_1_ chain of SLT-1 contains very few lysines (only 2 lysines located at its N-terminus) [45]. Therefore, arginine residues were projected to contribute to the creation of a positively charged surface on the A_1_ chain that may interact with the anionic aspartic acid residues of SDDDMGFGLFD. Arginines at positions 172, 176, 179, and 188 were mutated to alanines leading to A_1_ chain variants that were unable to interact in a yeast-2-hybrid experiment with SDDDMGFGLFD presented in the context of P2 (Figure 3A). These same SLT-1 A_1_ chain variants were also expressed and the resulting purified recombinant proteins were shown to have a decreased affinity or loss of binding to the conserved peptide SDDDMGFGLFD as determined by SPR (Figure 3B). In addition, increasing salt concentrations inversely correlated with binding of the wild-type A_1_ chain to the peptide as measured by SPR (Figure 3C). These findings coincide with recent evidence that shows that electrostatic interactions are critical for the binding of ricin to whole ribosomes and for the targeting of restrictocin to the sarcin-ricin loop [39], [40], [46]. Moreover, several surface-exposed hydrophobic or serine residues on the A_1_ chain in close proximity to the previously defined arginine cluster were mutated to alanine. Residues V191, F226, L233, and S235 were identified by Y2H as critical in maintaining the interaction with the conserved peptide SDDDMGFGLFD, the only docking site on the full length ribosomal protein P2 (Figure 4A). It was confirmed by SPR that residues V191, and L233 serve important roles in the interaction, whereas alanine mutations to residues F226 and S235 had no effect on binding (Figure 4B).

Structural data of a type I RIP, trichosanthin (TCS), and a type III RIP from maize supports our previous hypothesis and experimental data that charged and hydrophobic residues are important for the interaction with the C-terminus to occur [47], [48]. When the structure of SLT-1 A_1_ chain is compared to that of TCS it reveals the presence of a similar complementary surface-exposed groove theoretically predicted to involve residues R176, R179, and S235 of the A_1_ chain of SLT-1 [47]. Two of the three residues (R176 and R179) have been identified in this study as being essential for the interaction with the C-terminal peptide. Recent evidence has also been published highlighting the importance of R176 on the depurination activity of SLT-1 [49]. In addition, we have observed that V191, R172, R188, and L233 also contribute to the binding of the A_1_ chain to the conserved ribosomal peptide. However, the predicted A_1_ chain-peptide interaction appears to be unique when compared to the known structure of TCS in complex with the conserved peptide [47].

The SLT-1 A_1_ chain variants harboring R-A or hydrophobic mutations, which display striking decreased affinities for the ribosomal stalk peptide also show a reduction in their abilities to inhibit protein synthesis as measured by *in vitro* protein translation assays (Figure 5). These variants still retain their catalytic activity at high concentrations suggesting that a single substitution does not affect the overall three-dimensional fold of the A_1_ chain (Figure 5). These results suggest that depurination may still occur at high concentrations due to rRNA binding or that the pentavalent presentation of the conserved ribosomal peptide in the context of the intact ribosomal stalk still favors A_1_ chain docking to the stalk. Specifically, it may be sufficient for the A_1_ chain of SLT-1, and other related RIPs cytotoxic domains, to bind directly to rRNA since the depurination of the sarcin-ricin loop has been observed in protein-depleted ribosomes, although the depurination rate is remarkably reduced [19].

**Figure 6:**
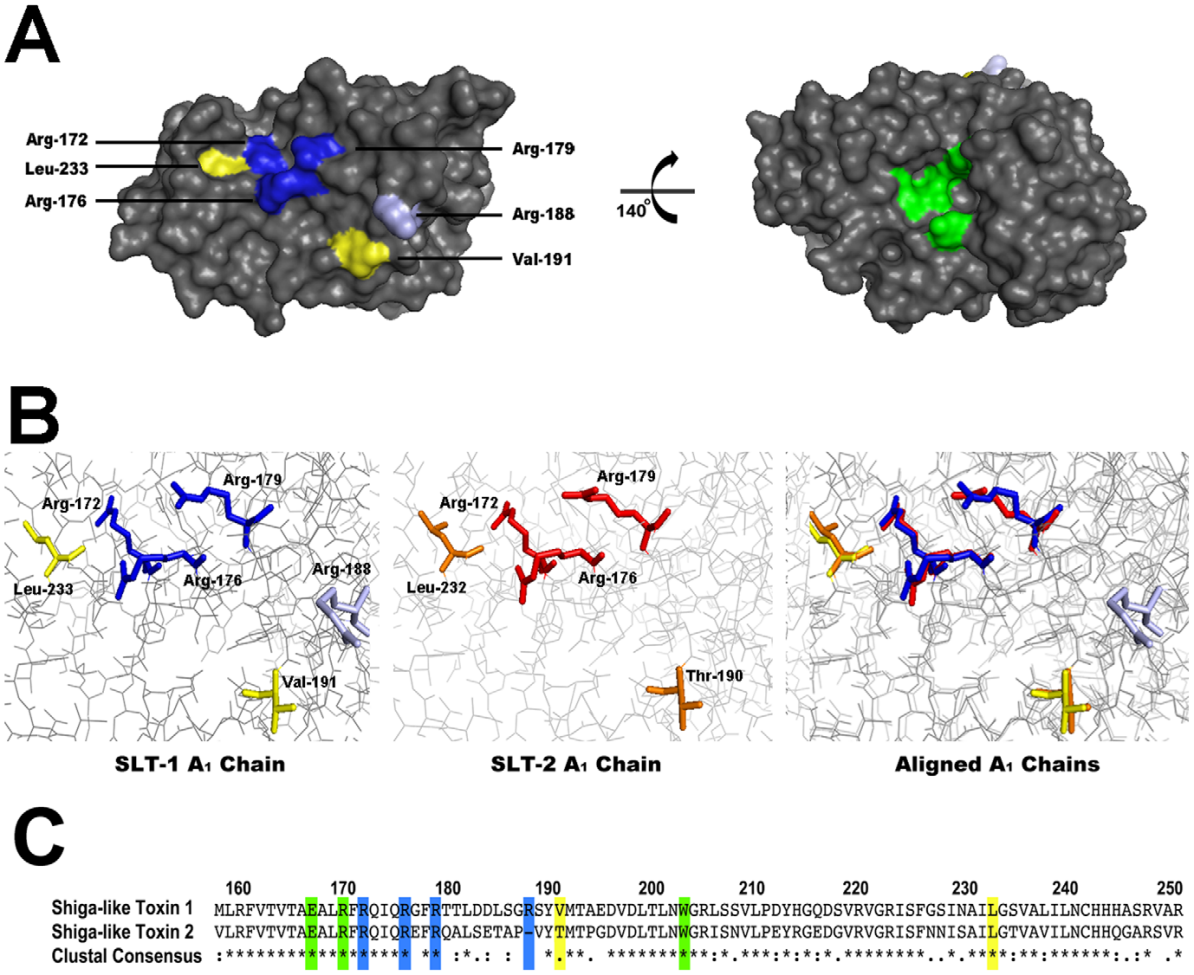
Primary and tertiary structural comparisons between SLT-1 and SLT-2 highlighting the conservation of important ribosomal stalk peptide contact sites. (A) *Left Panel* - Surface rendering of the SLT-1 A_1_ chain (PDB# 1DM0) depicting the cationic (blue) and hydrophobic (yellow) residues essential for optimal binding to the conserved stalk peptide SDDDMGFGLFD as well as Arg-188 (light blue) which has a modest effect on peptide binding. *Right Panel* – Structure as shown in the left panel rotated by 140°, highlighting the catalytic residues in green. (B) Three-dimensional stick structures of SLT-1 (left panel), SLT-2 (PDB# 1R4P; middle panel), and the structural alignment of the two toxins (right panel). Cationic residues are labeled in blue and red, while hydrophobic residues are labeled in yellow and orange for SLT-1 and SLT-2 respectively. (C) Primary amino acid sequence alignment of SLT-1 and SLT-2 within residues 158 and 250. Catalytic residues are highlighted in green and cationic and hydrophobic residues in blue and yellow, respectively. Surface and stick renderings and alignments were performed using the The PyMOL Molecular Graphics System (Version 1.3, Schrödinger, LLC), whereas amino acid sequences were aligned using BioEdit software [59].

This conserved ribosomal peptide represents a docking site for RIPs as it has been previously shown to interact with the catalytic domains of SLT-1, ricin, TCS, and maize RIP [15], [20], [47], [48]. It is therefore highly likely that SLT-2, an SLT-1 homologue that is produced by clinically more severe bacterial strains [50], would possess similar docking residues as we have shown for SLT-1. Even though the two catalytic domains share only a 55% homology based on amino acid sequence [51], it was observed that most of the surface residues important for the SLT-1-ribosomal peptide interaction were conserved on the surface of SLT-2 when the two toxins were aligned based on their respective tertiary structures (Figure 6). Specifically, five of the six residues shown to be important for the SLT-1 interaction (R172, R176, R179, V191, and L233) were conserved on SLT-2 (R172, R176, R179, Y189, and L232) (Figure 6, panels B and C). The only missing A_1_ chain interacting residue is arginine 188 of SLT-1, which is absent in SLT-2 (Figure 6, panels B and C). However, the A_1_ chain variant R188A displayed a catalytic activity comparable to that of wild type A_1_ chain (Figure 5).

This surface pocket and its affinity for the conserved C-terminal peptide may help explain why different RIP family members have the ability to bind the stalk conserved peptide and also why some RIPs such as pokeweed antiviral protein (PAP) do not require the stalk proteins for ribosome inactivation [52]. For example, the RIP saporin has a homologous tertiary structure to that of TCS, the A chains of SLT-1 and ricin, and was predicted to bind to the C-terminal tail of ribosomal stalk proteins [47]. Yet, we did not observe any measurable interactions between the C-terminal peptide SDDDMGFGLFD and the RIP saporin or the ribonuclease alpha-sarcin, which both target the sarcin-ricin loop, by either pull-down experiments, SPR, or by isothermal titration calorimetry (Figure S3). The A_1_ chain of SLT-1 was confirmed to interact with the C-terminal peptide by both isothermal calorimetry and pull-down experiments (Figure S3). Although the *on* and *off* rates for the toxin-peptide interaction could not be measured by SPR, the HiCaM-tethered peptide was able to interact with the toxin most likely due to the excess (10∶1) of the HiCaM-peptide used in the pull-down experiments in relation to the SLT-1 A_1_ chain. Thus, the identified surface on the SLT-1 A_1_ chain may therefore represent only one docking element that may not be generalized to the docking process of all RIPs.

Gastrointestinal disease outbreaks caused by infection with Shiga toxin-producing *E. coli* strains remain very common and suggest the need for post-infection therapeutic inhibitors [53], [54], [55]. The charged and hydrophobic surfaces mapped in this study provide a binding interface that is distinct from the catalytic site and are required for the full toxicity of SLT-1 *in vitro*. This structural information can be used for the generation of therapeutic inhibitors for both SLT-1 and SLT-2. For example, it has been shown that small molecule virtual-docking can be exploited to generate chemical compounds directed towards the catalytic domains of ricin, shiga toxins, and *Clostridium* botulinum neurotoxin [56], [57], [58]. These studies may provide molecular “leads” that may be more suited in terms of cell-permeability, affinities and *in vivo* stability than ribosomal stalk C-terminal peptide mimics as RIP antidotes.

In summary, the A_1_ chain of SLT-1 interacts transiently (K_d_∼13 µM with rapid *on* and *off* rates) with a short 11- amino acid conserved peptide located at the C-terminus of three ribosomal stalk proteins (P0, P1, and P2). The interaction involves both electrostatic and hydrophobic surfaces on both the A_1_ chain of SLT-1 and the ribosomal peptide SDDDMGFGLFD. Conversely, a cluster of positively charged arginine residues within the A_1_ chain in spatial proximity to a series of hydrophobic residues were defined as being critical for A_1_ chain binding to the peptide SDDDMGFGLFD and to inhibit protein synthesis *in vitro*. Thus, the catalytic A chain of Shiga toxins and other related RIPs may have evolved to interact rapidly and with low affinity to proteins constituting the ribosomal stalk in order to properly orient themselves towards their substrate, the sarcin-ricin loop.

## Supporting Information

Figure S1 **SDS-PAGE gel showing the relative purities of recombinantly expressed and purified SLT-1 A_1_ chain mutants.** Each SLT-1 variant was expressed and purified as described in the methods section. Purified wild-type SLT-1 A_1_ and point mutants were analyzed by SDS-PAGE and protein bands visualised by Coomassie blue staining. Numbers below each lane correspond to the purity of the major protein band (as a percentage) in relation to minor contaminating proteins as derived from densitometry measurements using the ImageJ software package.
(TIF)

Figure S2 **The SLT-1 A_1_ chain mutants F226A and S235A bind to the conserved C-terminal ribosomal peptide with similar affinity to wild-type SLT-1 A_1_.** Relative surface plasmon resonance (SPR) signals for the F226A and S235A SLT-1 A_1_ chain variants binding to immobilized synthetic SDDDMGFGLFD peptide were plotted as a function of SLT-1 A_1_ chain concentration. The calculated dissociation constants (K_d_) suggest that the F226A and S235A mutations in the A_1_ chain do not affect their affinity for the ribosomal stalk peptide SDDDMGFGLFD. Each point on the curve represents the average relative SPR signals from experiments performed in quadruplicate.
(TIF)

Figure S3 **The conserved peptide SDDDMGFGLFD interacts with the A_1_ chain of SLT-1 but may not be a generic contact site for all ribotoxins. (Top Panel)** Phenyl Sepharose bound HiCaM [60] fusion constructs (100 µg) displaying the C-terminal 7 amino acids (Lanes 4–5), 11 amino acids (Lanes 6–7), 17 amino acids (Lanes 8–9) of P1 and P2, or HiCaM alone (Lanes 2–3) were incubated briefly with 10 µg of SLT-1 A_1_ chain (Lane 1; Panel A), 20 µg saporin (Lane 1; Panel B), or 20 µg sarcin (Lane 1; Panel C) and separated on SDS-PAGE followed by Coomassie blue staining, as described previously [15]. The presence of a protein band in the thrombin cleavage (TC) lanes indicates an interaction and is only seen when the RIP A chain interacts with the final 11 or 17 residues of the conserved peptide. Legend: FT, column flow-through (unbound RIP); TC, thrombin-cleaved peptide. **(Lower Panel)** Synthetic peptide (starting with 500 µM) was titrated into a sample cell containing a 25 µM solution of degassed recombinant RIP and heat changes were measured using a VP-ITC (MicroCal Inc., Northampton, MA). The resulting calorimetric titration curves, minus the first injection of only 2 µl, were fitted using a single site binding model using the ORIGIN® software.
(TIF)

Table S1 **Primers used to construct expression vectors. Restriction endonuclease sites are underlined and amino acid substitutions are in bold.**
(TIF)
