# Supplementary figures and images for: Charged and Hydrophobic Surfaces on the A Chain of Shiga-Like Toxin 1 Recognize the C-Terminal Domain of Ribosomal Stalk Proteins
Source: PLoS One. 2012 Feb 15;7(2):e31191. doi: 10.1371/journal.pone.0031191 (PMC3280276; doi:10.1371/journal.pone.0031191)

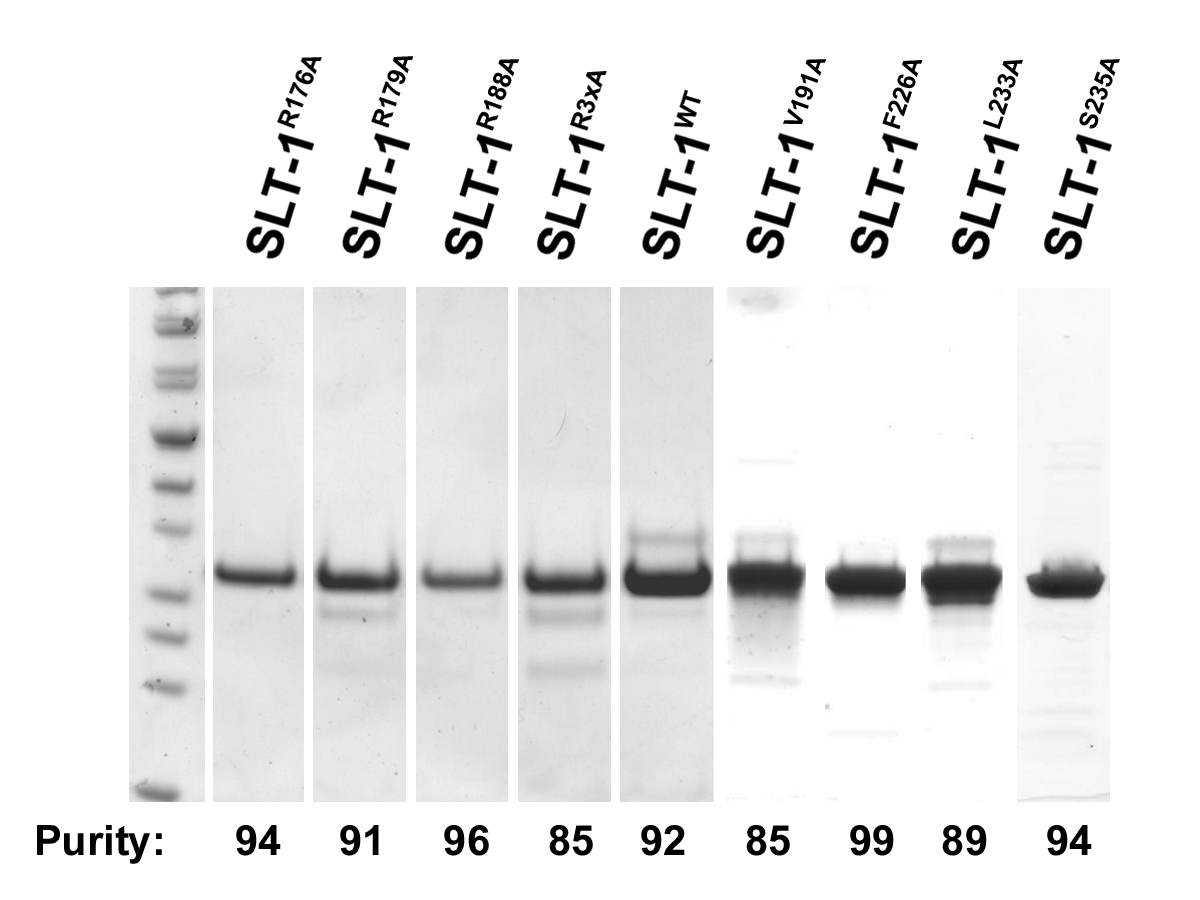

Supplement: Figure S1 — SDS-PAGE gel showing the relative purities of recombinantly expressed and purified SLT-1 A1 chain mutants. Each SLT-1 variant was expressed and purified as described in the methods section. Purified wild-type SLT-1 A1 and point mutants were analyzed by SDS-PAGE and protein bands visualised by Coomassie blue staining. Numbers below each lane correspond to the purity of the major protein band (as a percentage) in relation to minor contaminating proteins as derived from densitometry measurements using the ImageJ software package. (TIF) [file pone.0031191.s001.tif]

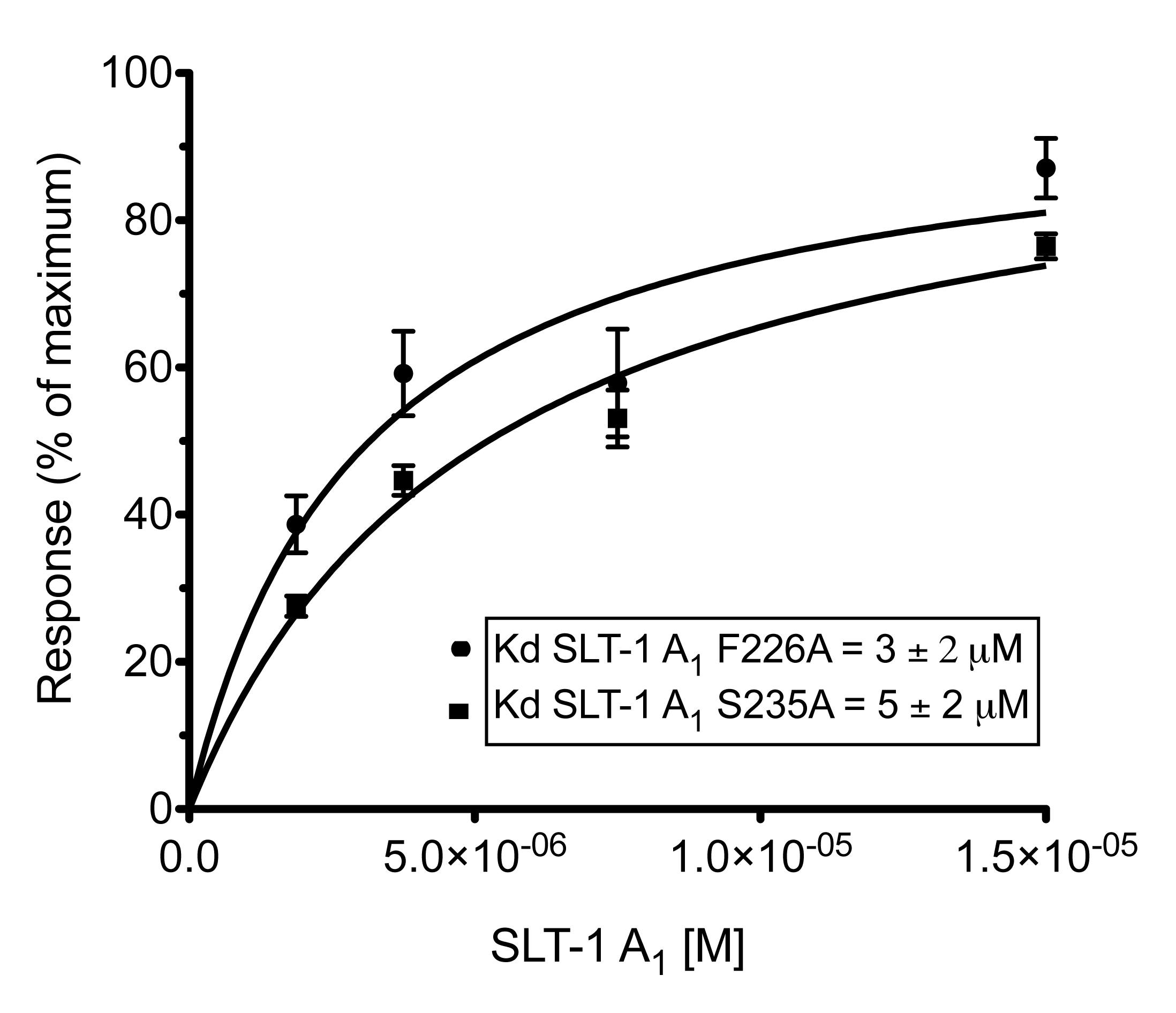

Supplement: Figure S2 — The SLT-1 A1 chain mutants F226A and S235A bind to the conserved C-terminal ribosomal peptide with similar affinity to wild-type SLT-1 A1. Relative surface plasmon resonance (SPR) signals for the F226A and S235A SLT-1 A1 chain variants binding to immobilized synthetic SDDDMGFGLFD peptide were plotted as a function of SLT-1 A1 chain concentration. The calculated dissociation constants (Kd) suggest that the F226A and S235A mutations in the A1 chain do not affect their affinity for the ribosomal stalk peptide SDDDMGFGLFD. Each point on the curve represents the average relative SPR signals from experiments performed in quadruplicate. (TIF) [file pone.0031191.s002.tif]

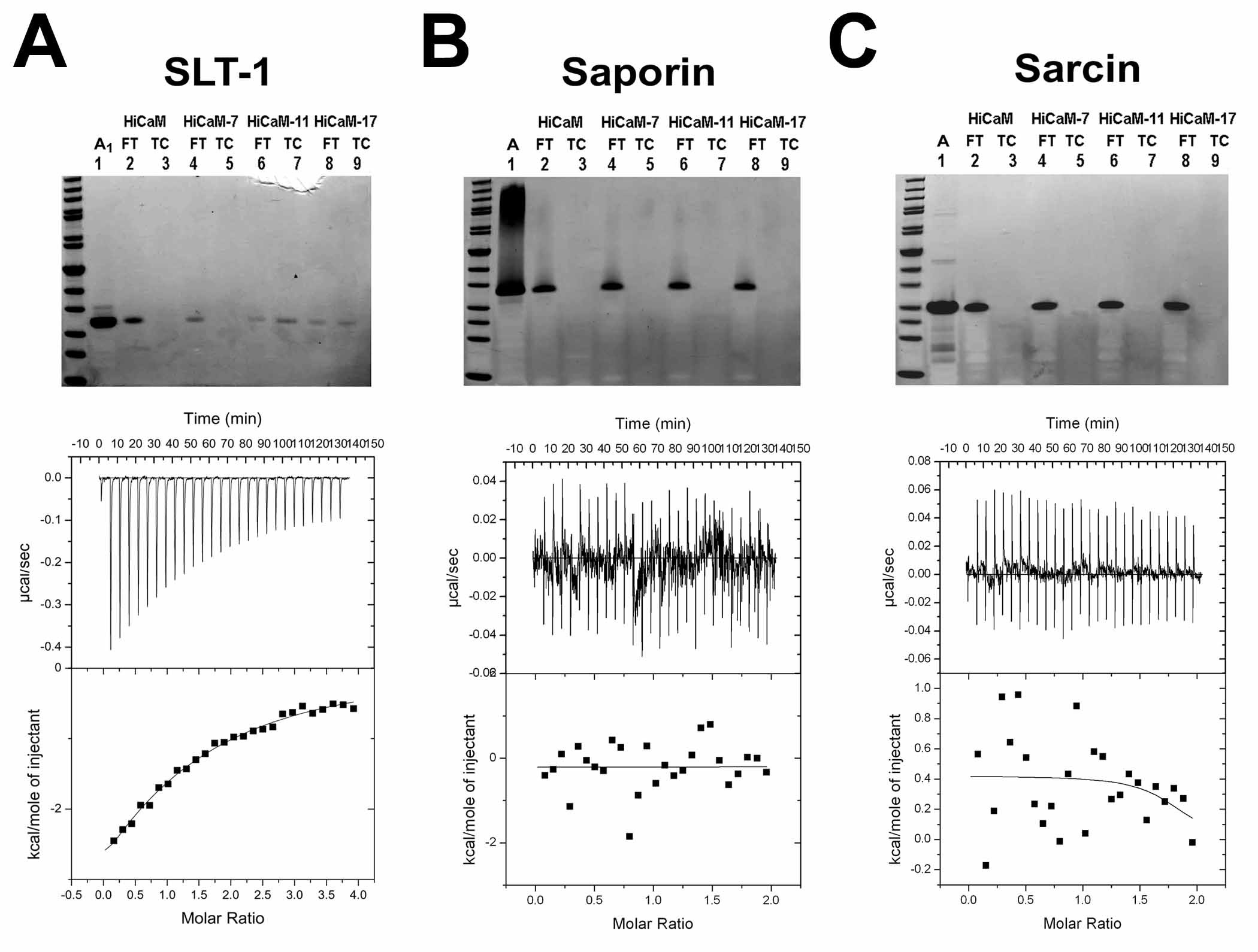

Supplement: Figure S3 — The conserved peptide SDDDMGFGLFD interacts with the A1 chain of SLT-1 but may not be a generic contact site for all ribotoxins. (Top Panel) Phenyl Sepharose bound HiCaM [60] fusion constructs (100 µg) displaying the C-terminal 7 amino acids (Lanes 4–5), 11 amino acids (Lanes 6–7), 17 amino acids (Lanes 8–9) of P1 and P2, or HiCaM alone (Lanes 2–3) were incubated briefly with 10 µg of SLT-1 A1 chain (Lane 1; Panel A), 20 µg saporin (Lane 1; Panel B), or 20 µg sarcin (Lane 1; Panel C) and separated on SDS-PAGE followed by Coomassie blue staining, as described previously [15]. The presence of a protein band in the thrombin cleavage (TC) lanes indicates an interaction and is only seen when the RIP A chain interacts with the final 11 or 17 residues of the conserved peptide. Legend: FT, column flow-through (unbound RIP); TC, thrombin-cleaved peptide. (Lower Panel) Synthetic peptide (starting with 500 µM) was titrated into a sample cell containing a 25 µM solution of degassed recombinant RIP and heat changes were measured using a VP-ITC (MicroCal Inc., Northampton, MA). The resulting calorimetric titration curves, minus the first injection of only 2 µl, were fitted using a single site binding model using the ORIGIN® software. (TIF) [file pone.0031191.s003.tif]

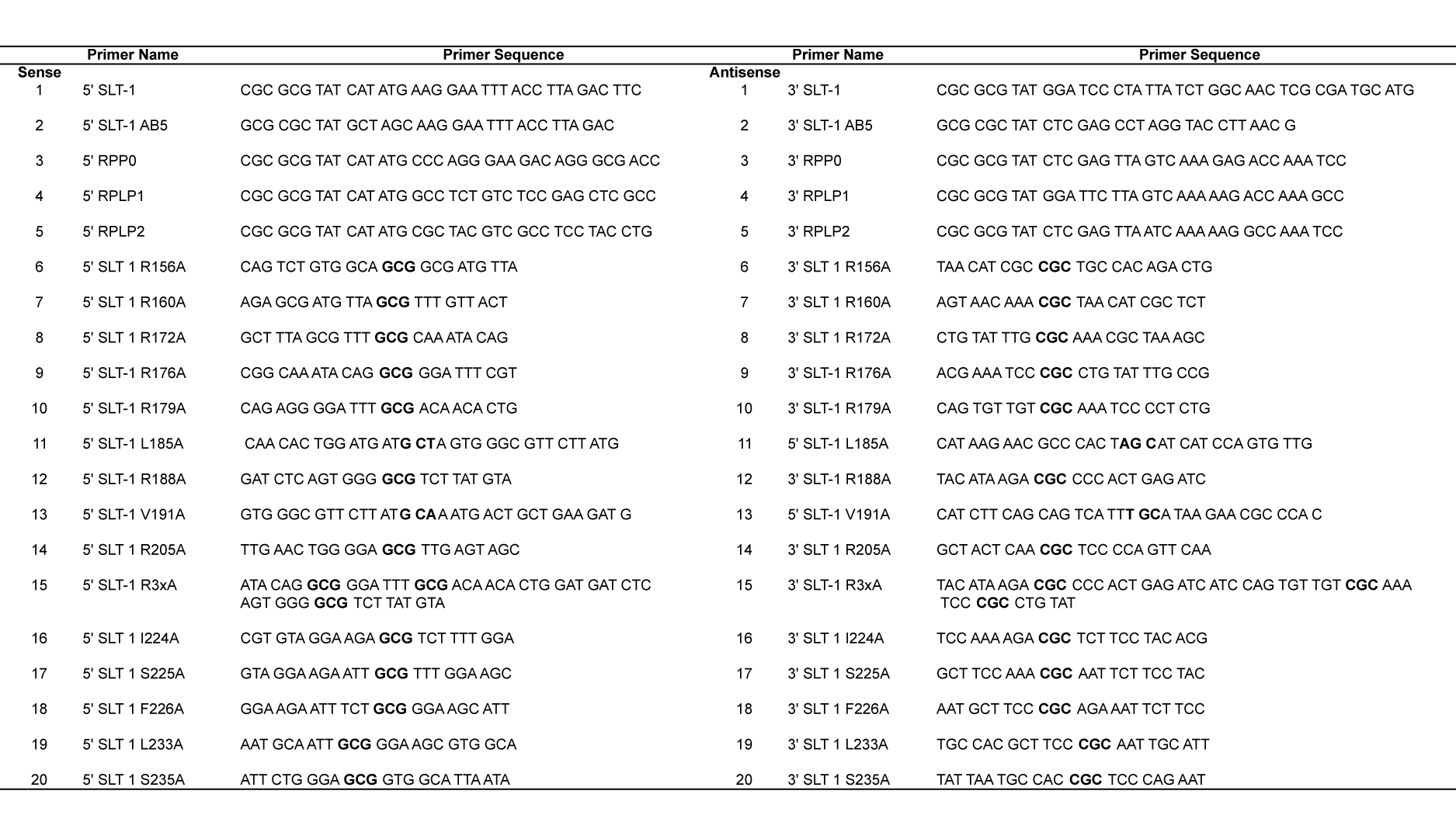

Supplement: Table S1 — Primers used to construct expression vectors. Restriction endonuclease sites are underlined and amino acid substitutions are in bold. (TIF) [file pone.0031191.s004.tif]
